# On a model-based approach to improve intranasal spray targeting for respiratory viral infections

**DOI:** 10.3389/fddev.2023.1164671

**Published:** 2023-05-30

**Authors:** Mohammad Mehedi Hasan Akash, Yueying Lao, Pallavi A. Balivada, Phoebe Ato, Nogaye K. Ka, Austin Mituniewicz, Zachary Silfen, Julie D. Suman, Arijit Chakravarty, Diane Joseph-McCarthy, Saikat Basu

**Affiliations:** ^1^ Department of Mechanical Engineering, South Dakota State University, Brookings, SD, United States; ^2^ Department of Biomedical Engineering, Boston University, Boston, MA, United States; ^3^ Joint Department of Biomedical Engineering, University of North Carolina–North Carolina State University, Chapel Hill, NC, United States; ^4^ Aptar Pharma, Congers, NY, United States; ^5^ Fractal Therapeutics, Cambridge, MA, United States; ^6^ Bioengineering Technology and Entrepreneurship Center, Boston University, Boston, MA, United States

**Keywords:** nasal drug delivery, respiratory transport, in silico modeling, computational fluid dynamics, experimental validation, intranasal spray, viral infections, nasal targets

## Abstract

The nasopharynx, at the back of the nose, constitutes the dominant initial viral infection trigger zone along the upper respiratory tract. However, as per the standard recommended usage protocol (“Current Use”, or CU) for intranasal sprays, the nozzle should enter the nose almost vertically, resulting in sub-optimal nasopharyngeal drug deposition. Through the Large Eddy Simulation technique, this study has replicated airflow under standard breathing conditions with 15 and 30 L/min inhalation rates, passing through medical scan-based anatomically accurate human airway cavities. The small-scale airflow fluctuations were resolved through use of a sub-grid scale Kinetic Energy Transport Model. Intranasally sprayed droplet trajectories for different spray axis placement and orientation conditions were subsequently tracked via Lagrangian-based inert discrete phase simulations against the ambient inhaled airflow field. Finally, this study verified the computational projections for the upper airway drug deposition trends against representative physical experiments on sprayed delivery performed in a 3D-printed anatomic replica. The model-based exercise has revealed a new “Improved Use” (or, IU) spray usage protocol for viral infections. It entails pointing the spray bottle at a shallower angle (with an almost horizontal placement at the nostril), aiming slightly toward the cheeks. From the conically injected spray droplet simulations, we have summarily derived the following inferences: (a) droplets sized between 7–17 *μ*m are relatively more efficient at directly reaching the nasopharynx via inhaled transport; and (b) with realistic droplet size distributions, as found in current over-the-counter spray products, the targeted drug delivery through the IU protocol outperforms CU by a remarkable 2 orders-of-magnitude.

## 1 Introduction

**FIGURE 1:**
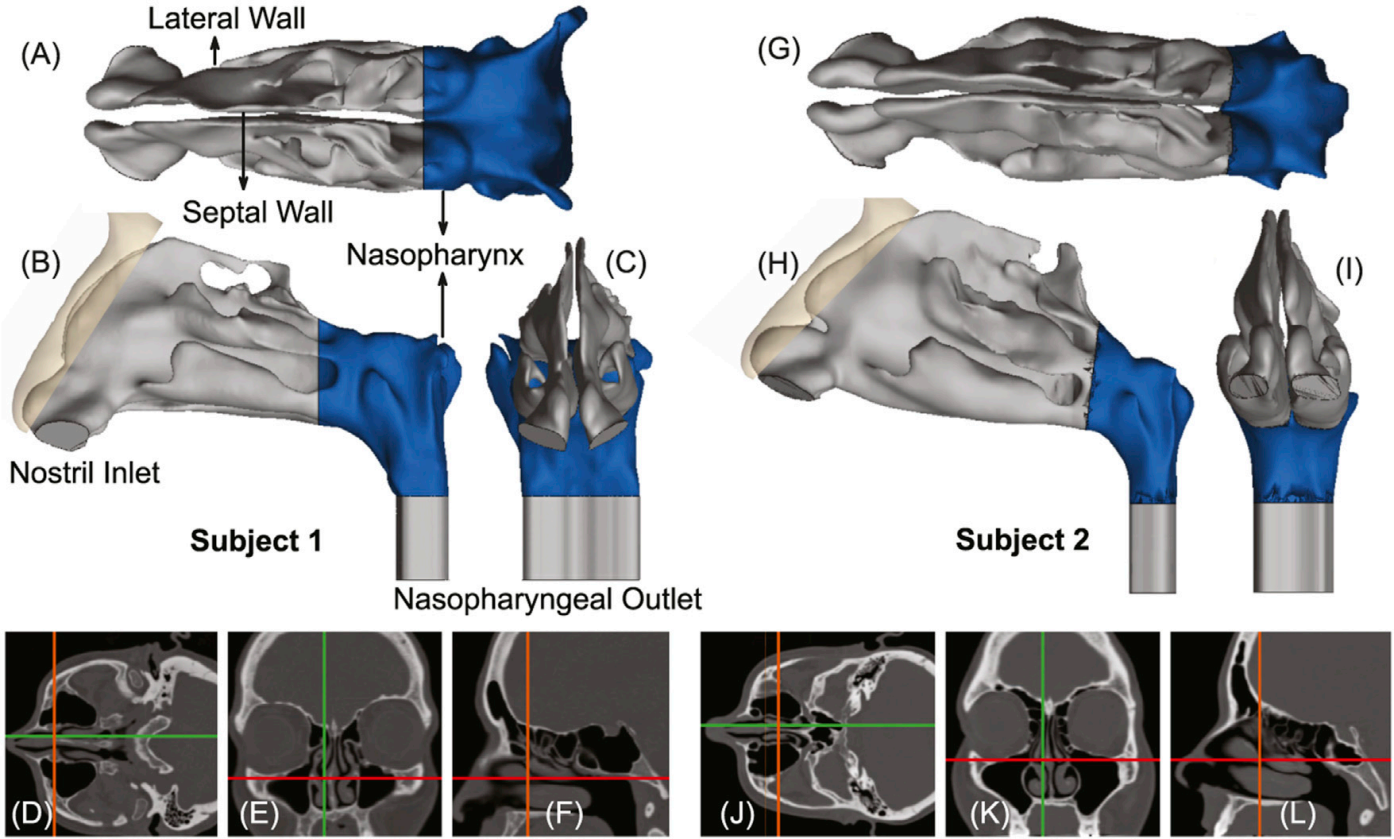
Panels **(A–C)**, respectively, show the axial, sagittal, and coronal views of the computed tomography (CT) based upper airway reconstruction in Subject 1. Panels **(D–F)** depict representative CT slices for the same subject. Therein, the green lines in **(D, E)** correspond to the location of the sagittal section shown in **(F)**; the orange lines in **(D, F)** correspond to the location of the coronal section shown in **(E)**; the red lines in **(E, F)** correspond to the location of the axial section shown in **(D)**. Panels **(G–I)** respectively show the axial, sagittal, and coronal views of the CT-based upper airway reconstruction in Subject 2. Panels **(J–L)** depict representative CT slices for the same subject. Therein, the green lines in **(J, K)** correspond to the location of the sagittal section shown in **(L)**; the orange lines in **(J, L)** correspond to the location of the coronal section shown in **(K)**; the red lines in **(K, L)** correspond to the location of the axial section shown in **(J)**. The nasopharynx has been marked in blue in panels **(A–C)** and **(G–I)**.

The global respiratory pandemic (COVID19-Dashboard, 2023) caused by the severe acute respiratory syndrome coronavirus 2 (SARS-CoV-2) has thrust the field of fluid mechanics into public eye, perhaps for the first time since the precarious era of 1960s′s space race (Mittal et al., 2020). Flow physics plays an essential role in almost every aspect of respiratory infections, none the more so than in targeted delivery of drugs to the infection hot-spots along the airway. Upper airway sites, specifically the ciliated epithelial cells that line the nasal passage, are rich in angiotensin-converting enzyme 2 (ACE2) surface receptors. Spike protein of SARS viruses binds to the ACE2 receptors to orchestrate cell intrusion (Wu et al., 2004; Basu A. et al., 2020). The anterior regions of the nasal cavity, however, present a relatively thick mucosal coating (Mittal et al., 2020), which provides a level of protection and prevention against viral invasions. Consequently, the nasopharynx at the back of the nasal cavity and situated immediately posterior to the convergence region of the two airway sides (see Figure 1)–has been identified (Hou et al., 2020; Matheson and Lehner, 2020; Basu, 2021) as the predominant trigger zone for infection onset owing to SARS-like airborne viral respiratory pathogens. Significant to note as well that the nasopharyngeal region contains the nasal-associated lymphoid tissue or NALT (Brandtzaeg, 2011), which provides a direct connection to the immune system. Accordingly, a targeted drug delivery modality geared toward intranasal vaccines and other prophylactic agents efficiently reaching the nasopharynx could be a key step forward in curbing respiratory viral transmissions and constitutes an underlying motivation for this study.

An early intervention method that can target the initial dominant infection site (i.e., the nasopharynx) is imperative for limiting asymptomatic transmission (Van Egeren et al., 2022) of the exhaled pathogenic particulates as well as for preventing systemic lower airway progression of the disease in a host, aggravating toward severe illness (He et al., 2020; Sungnak et al., 2020). Of critical interest here: based on the brisk pace at which lower airway infections often ensue after the emergence of initial symptoms, it has been conjectured that the nasopharynx also acts as the seeding zone for the spread of a respiratory viral disease to the lungs via lower airway aspiration of virus-laden boluses of nasopharyngeal fluids (Hou et al., 2020; Basu et al., 2022a; Chen et al., 2022). Such boluses would typically carry viral load far in excess of the virus-specific *infectious dose*, which, for example, has been shown to be on the order of a few hundred virions for SARS-CoV-2 (Basu, 2021; Ryan et al., 2021; Geddes, 2022). Another continuing concern is the mutation rate of a virus and how the nature of the fitness landscape renders it amenable to evolution, potentially resulting in more virulent strains (Pachetti et al., 2020; Van Egeren et al., 2021). A nasal spray–that can administer nasal hygiene products, intranasal vaccines, antiviral prophylactics and therapeutics–would address these concerns if it can efficiently deliver the pharmaceutics at the virus-affected upper airway sites, thereby reducing the risk of viral droplet/aerosol shedding (Yang et al., 2020; Giri et al., 2022) as well as mutation within the host (Valesano et al., 2021; Wang et al., 2021).

While the nasal sprays do provide a simple, yet robust, drug delivery modality, especially during the infection onset phase of respiratory viruses; with the choice comes at least two key *open* questions, *viz.* (a) which are the intranasally sprayed drug droplet sizes that would maximize targeted delivery at the initial dominant infection site, the nasopharynx? and (b) is there a way to revise the currently prescribed nasal spray usage protocols with prevalent spray products, to enhance the delivery of targeted drugs at the infected site?

This study addresses the above questions through experimentally verified computational fluid dynamics (CFD) modeling of the respiratory transport process in computed tomography (CT)-based anatomically realistic upper airway geometries. The related simulations replicate sprayed drug transmission against two different ambient inhalation rates, *viz.* 15 and 30 L/min; standing in respectively for steady relaxed and moderately heavy breathing conditions (Garcia et al., 2009). Preliminary findings pertaining to this work have been presented at the American Physical Society’s Division of Fluid Dynamics Annual Meeting in 2021 (Akash et al., 2021).

## 2 Materials and methods

### 2.1 Anatomic upper airway reconstruction

The *in silico* upper airway geometries used in this study were digitally reconstructed from de-identified medical-grade CT imaging data derived from two healthy test subjects. Subject 1 was a 61 year-old female and Subject 2 was a 37 year-old female. For subsequent experimental verification of the *in silico* findings, we have also used a 3D-printed solid anatomic replica of a 41 year-old male subject’s nasal cavity. The use of the archived and anonymized medical records was approved with exempt status by the Institutional Review Board (IRB) of the University of North Carolina (UNC) at Chapel Hill, with the requirement of informed consent waived for retrospective use in computational research.

In terms of imaging resolution, the CT slices of the airway cavities were extracted at coronal depth increments of 0.348 mm in Subject 1’s scans and 0.391 mm in Subject 2’s scans. Digitization of the anatomic airspaces was carried out on the image processing software Mimics Research v18.0 (Materialise, Plymouth, Michigan), using a radio-density delineation range of −1,024 to −300 Hounsfield units, and was complemented by clinically-monitored hand-editing of the selected pixels to ensure anatomic accuracy. The output STL (stereolithography) geometries were then spatially meshed on ICEM-CFD 2019 R3 (ANSYS Inc., Canonsburg, Pennsylvania) with minute volume elements. Therein, to confirm grid-independent solutions, established mesh-refinement protocols (Frank-Ito et al., 2016; Basu et al., 2017b) were followed such that each computational grid contained more than 4 million unstructured, graded, tetrahedral elements. To enable accurate tracking near tissue surfaces, further mesh refinement involved adding three prism layers at the cavity walls, with 0.1 mm thickness and a height ratio of 1.

### 2.2 Simulation of breathing transport and drug delivery

Inhalation parameters for gentle-to-moderate breathing conditions were numerically replicated at 15 and 30 L/min (Garcia et al., 2009). The lower flow rate commensurate with resting breathing is dominated by viscous-laminar steady-state flow physics (Basu et al., 2018; Farzal et al., 2019; Inthavong et al., 2019; Kimbell et al., 2019; Zhang et al., 2019; Basu S. et al., 2020). The higher flow rate for moderately heavy breathing (e.g., during sniffs), however, triggers shear-induced (Brown and Stewartson, 1969; Smith, 1986; Stremler and Basu, 2014; Basu and Stremler, 2017; Basu and Stremler, 2015; Stremler et al., 2020) flow separation from the tortuous cavity walls, resulting in turbulence (Longest and Vinchurkar, 2007; Doorly et al., 2008; Perkins et al., 2018; Hosseini et al., 2020). The latter was tracked through Large Eddy Simulation (LES), with sub-grid scale Kinetic Energy Transport Model (Baghernezhad and Abouali, 2010; Farnoud et al., 2020) accounting for the small-scale fluctuations. The computational scheme on ANSYS Fluent 2019 R3 employed a segregated solver, with SIMPLEC pressure-velocity coupling and second-order upwind spatial discretization. Solution convergence was monitored by minimizing mass continuity and velocity component residuals, and through stabilizing mass flow rate and static pressure at airflow outlets (see the nasopharyngeal outlet location in Figure 1). For the pressure gradient-driven laminar airflow solutions, the typical execution time for 5,000 iterations was 2–3 h with 4-processor based parallel computations operating at 3.1 GHz speed on Xeon nodes. Additionally, the LES computations each required a run-time of 1–2 days, for a pressure-driven simulated flow interval of 0.25 s, with a time-step of 0.0001 s. To realistically capture the continuum properties for inhaled warmed-up air transport inside the respiratory pathway, the air density and dynamic viscosity were set at 1.204 kg/m^3^ and 1.825 × 10^−5^ kg/m.s, respectively. However, note that the simulations did not incorporate any heat transfer effects.

Spray dynamics against the ambient airflow was tracked via Lagrangian-based inert discrete phase simulations with a Runge-Kutta solver, with localized droplet clustering along intranasal tissues obtained through numerically integrating the transport equations that consider airflow drag, gravity, and other body forces relevant for small particulates, e.g., the Saffman lift force, and by implementing a no-slip trap boundary condition on the cavity walls. Note that Brownian effects were neglected in view of the tracked droplet sizes. The drug formulation density was set to 1.5 g/mL, as a realistic estimate (Michael et al., 2001; Alfadhel et al., 2011). All simulations released monodispersed inert drug droplets ranging in diameters from 1–24 *μ*m, with 3,000 monodispersed inert droplets being released during each iteration. The droplets were introduced into the airspace as a solid-cone injection emanating from a single source point where the spray nozzle is located, mimicking the action of a nasal spray. Aptar Pharma’s VP7, a commerically produced pharmaceutical nasal spray pump, with its accompanying dimension properties, such as plume angle and initial spray velocity, was used as an initial point of reference for the cone injections (Pharma, 2022). The droplets were given a starting velocity of 10 m/s (Liu et al., 2011) and a total non-zero mass flow rate of 1 × 10^−20^ kg/s for the streams in the spray cone. The plume angle (i.e., the half-angle at the spray cone vertex) and the intranasal nozzle insertion depth were selected (Basu S. et al., 2020) to be 27.93° and 5 mm, respectively. Subsequently, by varying the spray direction–a new usage condition that would significantly augment droplet deposition at the target site was detected. See our earlier publications (Basu S. et al., 2020; Basu, 2021) for additional details on the numerical setup.

### 2.3 On how to hold the spray bottle

A key parameter for targeted delivery is the direction of the nasal spray axis, as the sprayed droplet trajectories are often inertia-dominated (Finlay, 2001; Basu et al., 2018; 2017a; Basu S. et al., 2020). Instructional ambiguities (Benninger et al., 2004; Kundoor and Dalby, 2011) point toward a lack of definitive knowledge on the best ways to use a nasal spray device, with package inserts accompanying different commercial spray products often offering somewhat contrasting recommendations. There is, however, a consensus that the patient should tilt her/his head slightly forward, while holding the spray bottle upright (Benninger et al., 2004; NIH, 2013). There is an additional clinical recommendation (Flonase, 2022) to avoid pointing the spray directly at the septum, which is the separating cartilaginous wall between the two sides of the nasal cavity. These suggestions were adopted in our standardization (Kimbell et al., 2018; Basu S. et al., 2020) of “Current Use” (CU) protocol for topical sprays. The digital airway models were inclined forward by an angle of 22.5°, and the vertically-placed upright (Benninger et al., 2004) spray axis was aligned closer to the lateral nasal wall, at one-third of the distance between the lateral side and septal wall. Finally, the spray bottle was placed at the nostril to penetrate 5-mm into the airspace, to conform with the package recommendations of commercial sprayers (NIH, 2013) for a “shallow” intranasal nozzle placement.

**FIGURE 2:**
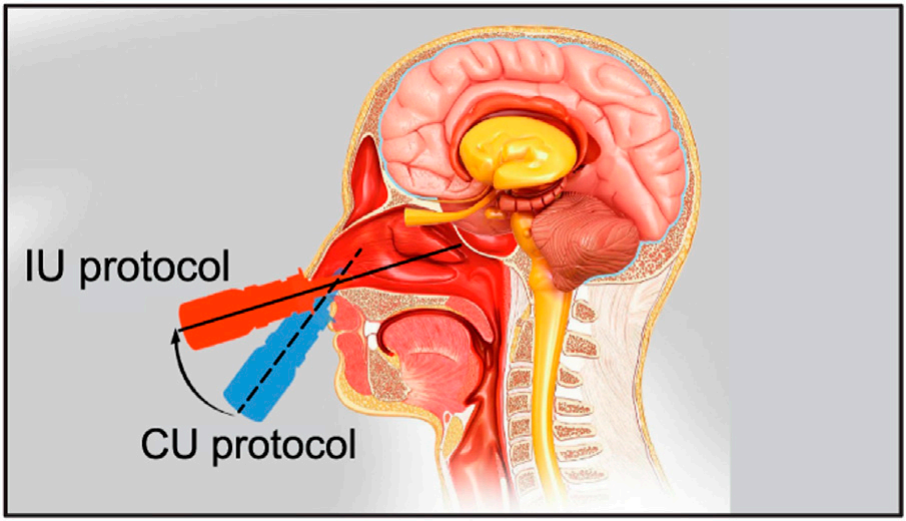
The schematic shows the two tested nasal spray usage protocols, *viz.* “Current Use” (or CU, represented by the dashed line) and “Improved Use” (or IU, represented by the solid line). Cartoon illustration is by the Dr. Ferrer Biopharma LLC (Hallandale Beach, FL) graphics design team.

**FIGURE 3:**
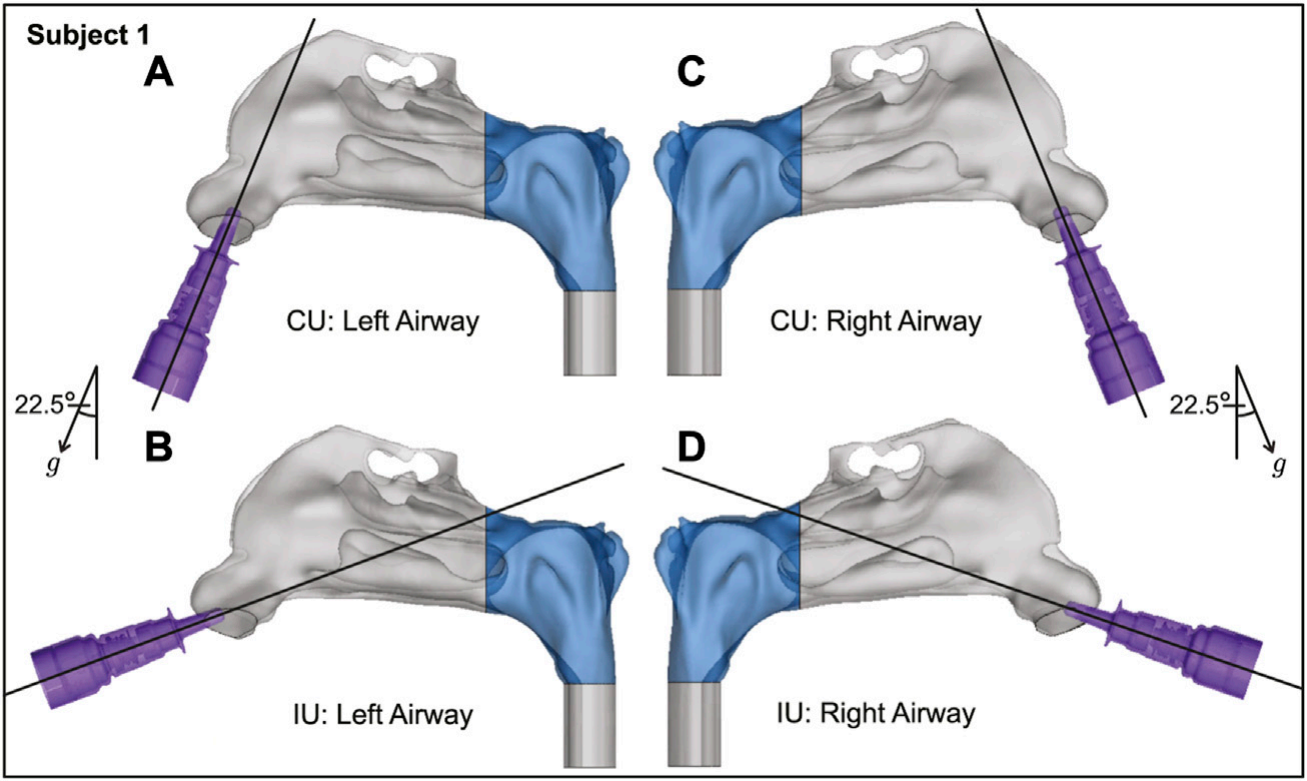
Spatial differences between the Current Use (CU) and Improved Use (IU) spray placement protocols, as visible sagittally in Subject 1. Nasopharynx is marked in blue, *g* points in the direction of gravity. Panels **(A, B)** show the left airway and panels **(C, D)** show the right airway in the same subject.

**FIGURE 4:**
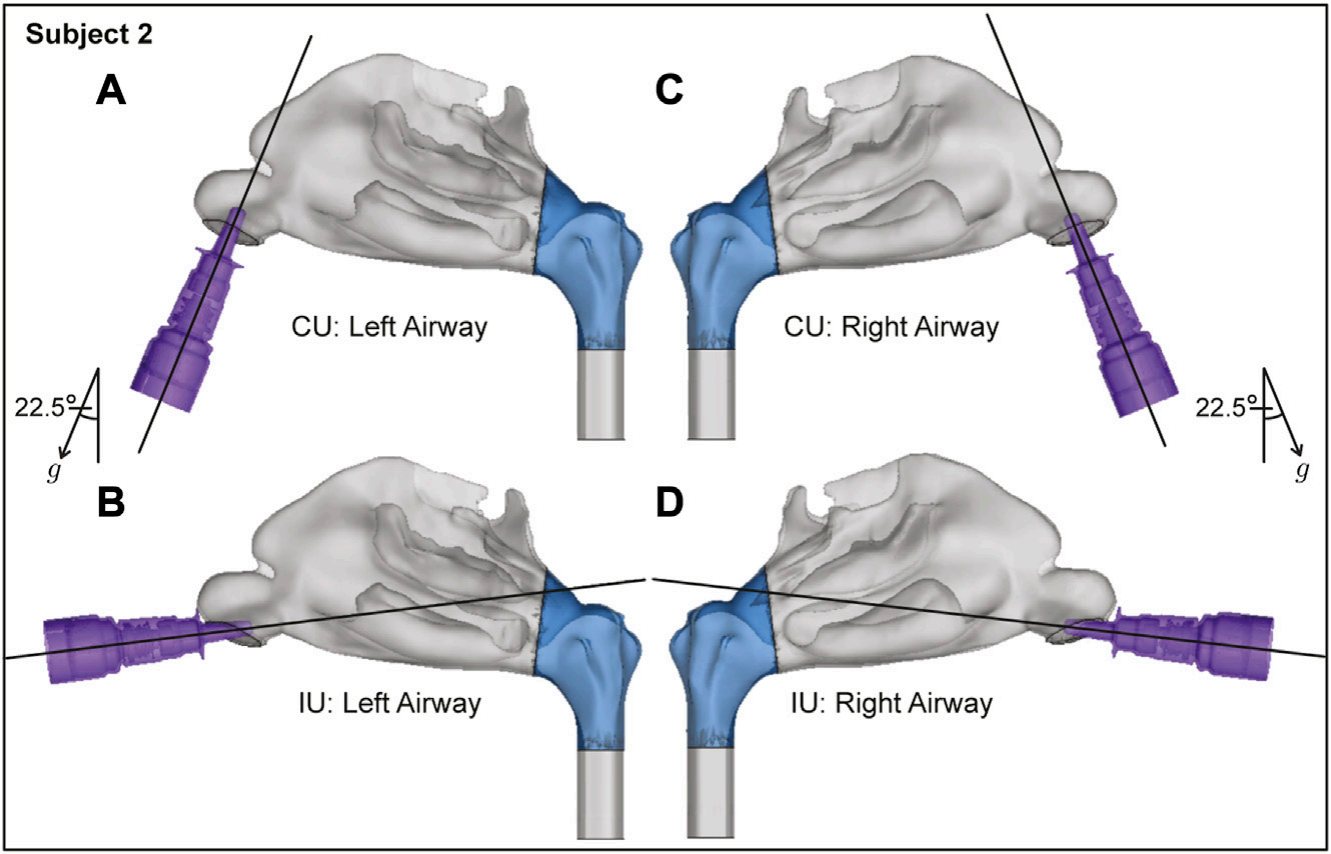
Spatial differences between the Current Use (CU) and Improved Use (IU) spray placement protocols, as visible sagittally in Subject 2. Nasopharynx is marked in blue, *g* points in the direction of gravity. Panels **(A, B)** show the left airway and panels **(C, D)** show the right airway in the same subject.

While the CU protocol is the accepted state-of-art technique for targeted drug delivery with nasal sprays, the key focus of this study was to re-examine the angle(s) at which the spray is administered relative to the nasal geometry (“spray direction”) to test alternate protocols that bear the promise to improve delivery of drugs at the nasopharyngeal infection site. Our earlier findings (Basu S. et al., 2020) showed that to target the clinical site of the ostiomeatal complex, or OMC (a key target site for corticosteroid-based topical therapeutic management for chronic rhinosinusitis (Farzal et al., 2019; Basu S. et al., 2020) and allergic rhinitis (Treat et al., 2020)), the spray axis should be oriented to pass through the OMC itself. The inertial motion of the sprayed particulates assists such a transport mechanism. Accordingly, to optimize the spray administration protocol in the current study, we oriented the nozzle such that the spray axis passes through the nasopharynx, and have named the strategy as “Improved Use”, or IU protocol. When determining the IU direction, it was important to satisfy three conditions as a way of ensuring the optimal placement of a nasal spray for drug release: (i) the extended spray axis for the IU protocol must intersect the nasopharynx; (ii) the spray axis must not cut through the septal wall to conform with clinical safety; and (iii) the axis should intersect the lateral wall in the posterior part of the nasal cavity. See the cartoonized Figure 2 for a broad-spectrum visual difference between the presently recommended CU and the to-be-tested IU protocols. Additionally, Figures 3, 4 depict the spatial distinctions in spray placement between the IU and CU protocols, in the two test subjects, as visible from the sagittal perspective.

### 2.4 Tolerance sensitivity analysis

Once the IU for an airway reconstruction was determined (following guidelines outlined in Section 2.3), an axis perturbation-based tolerance sensitivity study was performed to assess how far the user could deviate from the determined IU spray direction and still get comparable regional drug deposition results, or in other words how robust (or, on the contrary, user-sensitive) the chosen IU direction really is.

To generate the new perturbed axes in the *in silico* space, a 1-mm radius circle was created perpendicular to the perturbed direction either 5-mm or 10 mm away from the central point on the nostril plane of each model. The two different distances were chosen in order to test the sensitivity of the results at different perturbation levels. The 5 mm method was performed on the left nostril of the subjects, while the 10 mm method was performed on the right nostril. Five peripheral points equidistant from each other were then selected on the circle created. The axis formed between the centroid point on the nostril plane and the peripheral point on the circle determined the new perturbed direction (PD). In all, five additional perturbed spray axis directions were created for each nostril, henceforth referred to as PD 1–5. For each new perturbed direction, the injection point was selected by measuring 5 mm from the centroid on the nostril plane, toward the nasopharynx. Each new identified PD axis satisfied the criteria developed to identify the IU direction, as described in Section 2.3, and drug delivery simulations were performed following the methods laid out in Section 2.2. The results of the tolerance simulations were analyzed for congruity using Pearson’s correlation coefficient.

### 2.5 Experimental verification of computationally predicted spray performance

**FIGURE 5:**
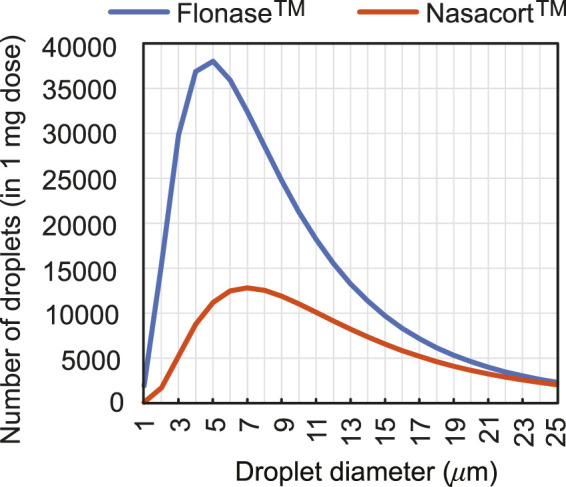
Observed count distribution of droplet sizes in 1-mg sprayed mass from over-the-counter Flonase™ (Fluticasone Propionate) and Nasacort™ (Triamcinolone Acetonide) spray products, over the test size range of ∼ 1–24 *μ*m used for *in silico* tracking. Note that rigorous numerical testing for droplets > 24 *μ*m clearly show (Basu S. et al., 2020; Basu, 2021) that they would mostly deposit along the anterior nasal cavity and will largely miss the posterior target site of the nasopharynx.

To extrapolate to real-world spray performance that could be projected from the *in silico* framework, we linked the computationally predicted nasopharyngeal droplet deposition efficiencies with the size distribution of droplets (see Figure 5) in two existing over-the-counter spray products–thus assessing the expected deposition at the nasopharynx with a typical nasal spray. Specifically, measured distributions for Flonase™ (Fluticasone Propionate) and Nasacort™ (Triamcinolone Acetonide), both of which are commonly prescribed medications that are commercially available, were used. Four units of each product were tested at Next Breath, an Aptar Pharma company (Baltimore, MD, United States). The team measured the plume geometry through a SprayVIEW^®^ NOSP, which is a non-impaction laser sheet-based instrument. With the droplet sizes in a spray shot following a log-normal distribution, the droplet size distribution (where droplet diameters are represented by *x*) can be framed as a probability density function (Cheng et al., 2001):

$$m\left(x\right)=\frac{1}{\sqrt{2\pi }x\ln\sigma_{g}}\exp\left[-\frac{{\left(\ln x-\ln x_{50}\right)}^{2}}{2{\left(\ln\sigma_{g}\right)}^{2}}\right].$$ (1)

Here the mass median diameters (Finlay, 2001) for Flonase™ and Nasacort™ were respectively: *x*_50_ = 37.16 *μ*m and 43.81 *μ*m; the corresponding geometric standard deviations were respectively: σ_*g*_ = 2.080 and 1.994. The latter statistically quantifies the measured range of the droplet size data, while the *x*_50_ marks the diameter such that 50% of the spray mass is in droplets smaller than *x*_50_. Note that the measurements were also collected with and without a saline additive in the sprayer, with the tests returning similar droplet size distributions. The reader is referred to our previous publications (Basu et al., 2018; Basu S. et al., 2020) for additional details.

In order to test the extensibility of the computational predictions derived for actual sprays, we subsequently conducted 20 runs of physical spray experiments with 10-mL boluses (for measurable posterior deposits) of dyed water-based solutions injected through a 3D-printed anatomically realistic airway cavity of a different subject, Subject 3 (a 41-year old male; the corresponding imaging data had a CT-slice resolution of 0.352 mm). Printing of the related anterior soft plastic part on a Connex3™ 3D printer was carried out using a polymer ink-jetting process on Tangogray FLX950 material, approximately mimicking the material properties of the external nares and the internal tissues and cartilages. The 3D-printed cavity extent terminated just before the nasopharynx, thereby allowing us to measure the outflow volume of administered solution that would reach the nasopharyngeal walls. During the experiments, the 3D cast was clamped and an adjustable angle hinge connector of diameter 0.75 in. was used to precisely fix the injector device to replicate the CU protocol (see Section 2.3). To recreate the IU protocol, the injection was administered horizontally (as much as possible) with the spray nozzle inserted at a shallow depth of 5 mm inside the airspace. Any discharge from the front of the nose was collected separately to ensure that it did not contaminate the measurement of the penetrating solution. The reader may briefly check Figures 9C, D for photographic representations of the 3D-printed soft nose used in the experiments. Additionally, for a review of prior work on the use of 3D-printed anatomic casts for nasal drug delivery studies, see Williams and Suman (2022).

## 3 Results

### 3.1 Improved orientation of the spray axis for effective targeting

**FIGURE 6:**
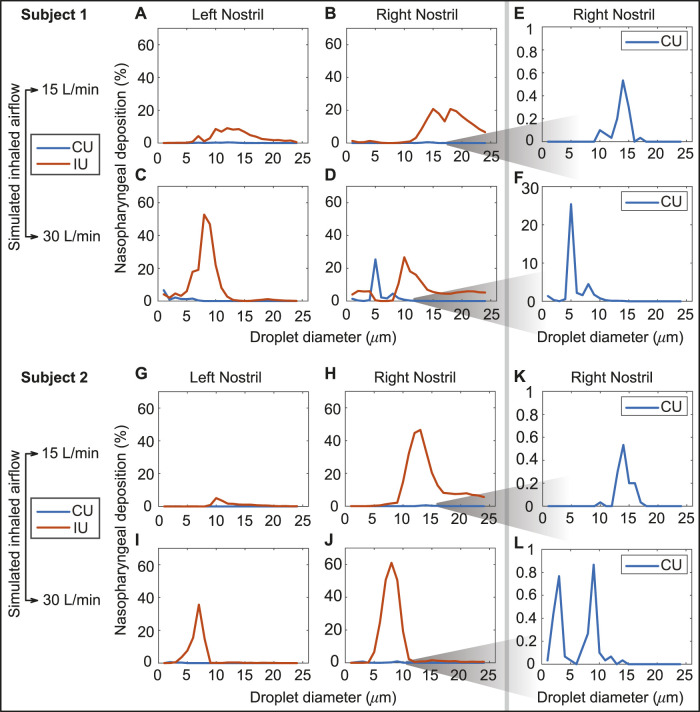
Panels **(A**–**D)** show the comparison of the regional deposition trends at the nasopharynx of Subject 1, for the IU and CU protocols, with monodispersed conical injections. The row comprising **(A**, **B)** are for 15 L/min inhalation; the row with **(C, D)** are for 30 L/min inhalation. Panels **(E, F)** depict the representative zoomed-in trends for nasopharyngeal deposition with the CU protocol, on administering the spray through the right nostril of Subject 1. Similarly, panels **(G, J)** show the comparison of the regional deposition trends at the nasopharynx of Subject 2, for the IU and CU protocols. The row comprising **(G, H)** are for 15 L/min inhalation; the row with **(I, J)** are for 30 L/min inhalation. Panels **(K,** **L)** depict the representative zoomed-in trends for nasopharyngeal deposition with the CU protocol, on administering the spray through the right nostril of Subject 2. The IU trend lines are marked in red; the CU trend lines are in blue. The reader should note the abbreviated vertical range on the **(E, F, K, L)** plots, prompted by the 2 orders-of-magnitude smaller deposition efficiency with CU.

Airflow and droplet transport have been simulated for spray nozzle placement at the left and right nostrils of Subjects 1 and 2 (see Section 2.1 for details on the test subjects), under two standard inhalation rates (15 and 30 L/min), for drug droplet diameters 1–24 *μ*m, and for spray directions as per the “Current Use” (or, CU) and “Improved Use” (or, IU) protocols. See Figure 2 for the respective spray usage protocol visuals, and also Section 2.3. In all eight cases, the IU direction of the spray axis results in higher deposition at the nasopharynx in comparison to the CU protocol (see Figure 6). For instance, if we examine the deposition trends for spray administration through the right nostril of Subject 2 for the laminar regime inhalation (i.e., at 15 L/min), the peak nasopharyngeal deposition for IU is 46.5% for 13 *μ*m drug droplets (Figure 6H), while the peak deposition for CU is only 0.53% for 14 *μ*m drug droplets (see again Figure 6H and the corresponding zoomed-in visual for the CU delivery trends in Figure 6K). The nearly hundred-fold increase in targeted deposition is remarkable and is achievable simply by re-orienting the spray axis from CU to IU.

### 3.2 Assessing sensitivity to IU perturbations

**FIGURE 7:**
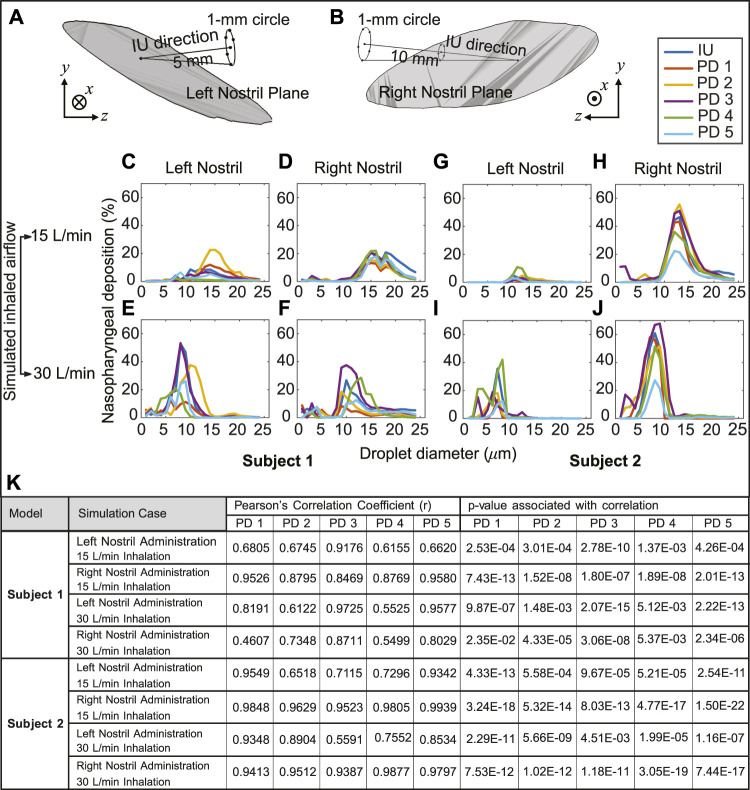
Panels **(A, B)** illustrate the *in silico* detection of the perturbed spray directions (PD), deviating slightly from the IU axis. The direction vectors are from the centroid of the nostril plane to the points lying on a 1-mm circle that is 5 mm and 10 mm (respectively for the left and right nostril placement) from the nostril plane centroid (see Section 2.4 for related details). Panels **(C–F)** for Subject 1 and panels **(G–J)** for Subject 2 compare the respective nasopharyngeal deposition trends for PD 1–5 directions, with respect to that of the “Improved Use” (IU) protocol. The top row is for 15 L/min inhalation; the bottom row is for 30 L/min inhalation rate. Clustering of the plots signifies robustness of the IU usage parameters; in other words, the IU protocol is satisfactorily insensitive to user subjectivities **(K)** Statistical tests are performed to check the correlation between the regional deposition efficiencies (for the discrete drug droplet sizes 1–24 *μ*m) at the nasopharynx for the perturbed spray directions (i.e., PD 1–5), when compared to the nasopharyngeal deposition efficiencies for the same droplet sizes with the IU protocol. The tabulated data includes the Pearson’s Correlation Coefficients (and associated *p*-values, with *α* = 0.05).

The variation of the nasopharyngeal deposition percentages over the assessed droplet size range (1–24 *μ*m) was compared between that of the IU protocol and each of the perturbed direction (PD) data, *viz.* PD 1–5. The PD spray orientations were obtained by slightly perturbing the IU direction; see Section 2.4 for details. Pearson’s correlation coefficient was comfortably greater than 0.5 for nearly every such comparison (see Figure 7), showing a high degree of linearity between the perturbed directions and the IU protocol in terms of the ranked order of the nasopharyngeal deposition efficiencies exemplified by the tested spray droplet sizes. Moreover, the *p*-value associated with each correlation was much lower than the significance level of ∼ 0.05. This indicates that there is a statistically significant correlation between the simulation results on the targeted nasopharyngeal drug delivery for the IU and the perturbed directions. Physically, the satisfactory correlation between IU and PD 1–5 establishes the robustness of the IU spray protocol to user subjectivities.

### 3.3 Verification of optimal droplet sizes through scaling analysis

The droplet size ranges that registered peak nasopharyngeal deposition under each inhalation condition were further analyzed and validated for reliability, through a Stokes number-based scaling analysis (Balachandar, 2009). The Stokes number (St), a ratio of the particle (droplet) response time to the ambient fluid (air) characteristic time scale is mathematically defined as (Finlay, 2001)

$$St=\frac{U\;\rho_{D}\;D^{2}\;C_{c}}{18\;\mu \;d},$$ (2)

where *U* for the present system is the airflow rate divided by flux area, $\rho_{D}$ is the material density of the inhaled droplets, D is the droplet diameter, *C*_*c*_ is the Cunningham slip correction factor, *μ* is the dynamic viscosity of the ambient medium (i.e., air), and *d* represents the characteristic diameter of the flux cross-section. Now, with all other flow and morphological parameters staying invariant, Eq. 2 directly leads to the following scaling law:

$$\frac{D_{2}}{D_{1}}=\sqrt{\frac{Q_{1}}{Q_{2}}}.$$ (3)

Herein $\left(Q_{i},D_{i}\right)$ are different inhaled airflow rate and sprayed droplet size pairings. Let us now consider a representative example, say the right nostril spray administration in Subject 2. For at least 2% nasopharyngeal deposition, the computationally predicted ideal droplet size range during 30 L/min inhalation is $\left[D_{\text{min}},\;D_{\text{max}}\right]=\left[5,\;11\right]\;\mu$ m. Eq. 3 can consequently help us to project the corresponding ideal size range at the lower inhalation rate of 15 L/min. If the to-be-projected droplet size range that would generate peak nasopharyngeal deposition during the 15 L/min inhalation is represented by $\left[D_{\text{min}}^{\prime },\;D_{\text{max}}^{\prime }\right]$ in *μ*m, then

$$\frac{D_{\text{min}}^{\prime }}{5}=\frac{D_{\text{max}}^{\prime }}{11}=\sqrt{\frac{30}{15}}.$$ (4)

**FIGURE 8:**
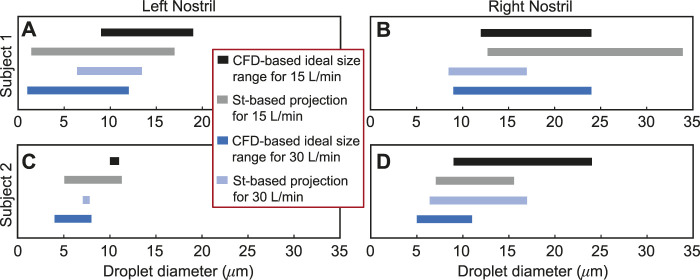
Panels **(A, B)** for Subject 1 and panels **(C, D)** for Subject 2 visually depict the Stokes number (St)-based projections of droplet size ranges for at least 2% targeted deposition at the nasopharynx. The directional change of the St-projected ranges along the number scale agrees with the corresponding CFD-based “ideal” droplet size ranges in all the test cases, except in one trivial outlier: see panel **(B)**, where the maximum ideal size limits at both 15 and 30 L/min are 24 *μ*m; the St-projected maximum ideal droplet size for 30 L/min is, however, 33.94 *μ*m. See Section 3.3 for a representative discussion for the data reported in **(D)**. Included in the inset is the color scheme for the plotted data in all four panels.

This results in $D_{\text{min}}^{\prime }=7.07\;\mu$ m and $D_{\text{max}}^{\prime }=15.56\;\mu$ m. Despite the simplicity of this scaling analysis, the computationally identified range 9–24 *μ*m for the same breathing conditions hence follows the same trend on the number scale, in terms of the respective directional variations from the extremal limits defined by $\left[D_{\text{min}},\;D_{\text{max}}\right]$ . Figure 8D visually illustrates this specific example; see the remaining panels in Figure 8 for all the other test cases. The directional change of the extremal limits for the St-projected ideal droplet size ranges remarkably agrees with the corresponding CFD-based size ranges in all cases, except in one trivial outlier: see panel (B) in Figure 8 for Subject 1’s right nostril, there the droplet size limits for at least 2% nasopharyngeal deposition with both 15 and 30 L/min inhalation rates are 24 *μ*m (an artifact resulting from the numerically tested droplet size range of 1–24 *μ*m); the St-projected maximum ideal droplet size for 30 L/min, however, comes out to be 33.94 *μ*m.

### 3.4 Generic ideal droplet size range for targeted nasopharyngeal delivery

Droplet diameter range of 7.375–16.625 *μ*m, or more practically ∼ 7–17 *μ*m, is found most conducive for targeted nasopharyngeal delivery with the IU spray protocol, considering a 2% cut-off for deposition efficiency of the tracked monodispersed droplet cluster of each size. The limits of the generic ideal size range are obtained by respectively calculating the mean of the CFD-predicted minimum and maximum droplet diameters plotted in solid black and dark blue in Figure 8; the averaging incorporated the droplet size data from all the eight test cases. It is, however, important to note that a dominant proportion of droplets (or, aerosols) that are smaller than 10 *μ*m can bypass the nose and deposit in the lungs (Crowder et al., 2002; Chakravarty et al., 2022; Darquenne et al., 2022). From a regulatory standpoint, this may constitute a risk and the US Food and Drug Administration (FDA) accordingly monitors the percentage of droplets smaller than 10 *μ*m for safety reasons.

### 3.5 Comparison of the *in silico* findings to physical experiments

**FIGURE 9:**
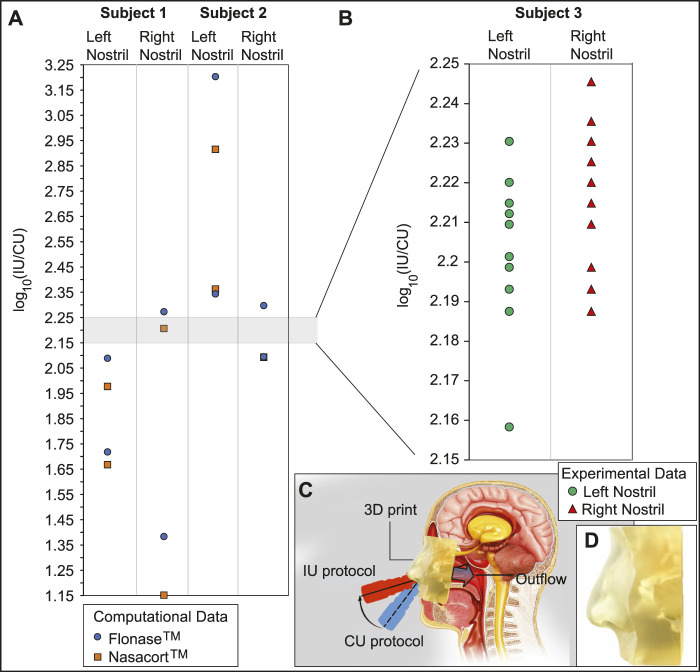
Experimental verification: Panel **(A)** shows the order-of-magnitude of IU-induced improvement in drug mass deposits at the nasopharynx of Subjects 1 and 2 (compared to the CU delivery numbers), when considering the droplet size distribution in each administered shot of two common over-the-counter spray products: Flonase™ and Nasacort™. Panel **(B)** shows the measurements from a set of physical experiments with sprayed watery solution in a different Subject 3. As an indicator for agreement between the computational and experimental projections, the vertical range in **(B)** is a medial subset of that in **(A)**. Note that several data-points roughly superimposed over each other, in both **(A, B)**. Panel **(C)** presents a cartoon of the experimental setup. A separate inset visual for the 3D-printed soft nose, with realistically pliable external nares, is shown in panel **(D)**. Underlying cartoon illustration in panel **(C)** has been prepared by the Dr. Ferrer Biopharma LLC (Hallandale Beach, FL) graphics design team.

Panel (A) in Figure 9 portrays the order-of-magnitude improvement in targeted drug deposition at the nasopharynx (with the IU protocol over the CU protocol), when taking into the account the droplet size distributions (Basu et al., 2018; Basu S. et al., 2020) in actual over-the-counter spray products, *viz.* Flonase™ and Nasacort™, in an administered shot. See Section 2.5 for the related study methods. Considering all the test cases, the average IU-over-CU improvement for the two chosen spray products, as projected from the CFD simulations, was 2.117 orders-of-magnitude, with a standard deviation of 0.506. The physical experiments in a new Subject 3 (presenting an anatomy distinct from that in Subjects 1 and 2, see Section 2.5) reveal a comparable mean improvement in nasopharyngeal delivery, by 2.215 orders-of-magnitude, with a standard deviation of 0.016. Panel (B) in Figure 9 plots the experimental measurements. The conformity on targeted delivery improvement between the computations and the representative physical experiments lends support to the implemented *in silico* framework.

### 3.6 Estimation for active pharmaceutical ingredient (API) delivery for sample over-the-counter spray products

To illustrate the practicality of our modeling approach in assessing drug therapeutic efficacy, let us now consider the experimental test data for the spray products reported in Section 2.5. The averaged estimates for the spray weight administered from each pump of a spray were 104.51 mg for Flonase™ (Fluticasone Propionate) and 97.64 mg for Nasacort™ (Triamcinolone Acetonide). Based on our simulation data and by imposing the droplet size distribution measured for Flonase™, the mean nasopharyngeal delivery during each spray pump was 1.9187 mg for the IU protocol and 0.0495 mg for the CU protocol. Subsequently, assuming an API concentration of 50 mcg/100 mg of formulation (DailyMed, 2023) results in 0.96 mcg API delivery at the nasopharynx during the IU protocol, with direct inhalation. The corresponding number for the CU protocol with Flonase™ is 0.025 mcg, hence remarkably lower than the IU performance. Subsequently, with the droplet size distribution for Nasacort™, our simulations result in 1.8450 mg mean nasopharyngeal delivery during each spray pump with the IU protocol. In comparison, the corresponding number with the CU protocol is 0.0482 mg. Consequently, for Nasacort™ which presents an API concentration of 55 mcg/110 mg of formulation (Drugs.com, 2023), the mean API mass delivered at the nasopharynx through direct inhalation would be 0.92 mcg for IU and 0.024 mcg for CU.

## 4 Discussion

**FIGURE 10:**
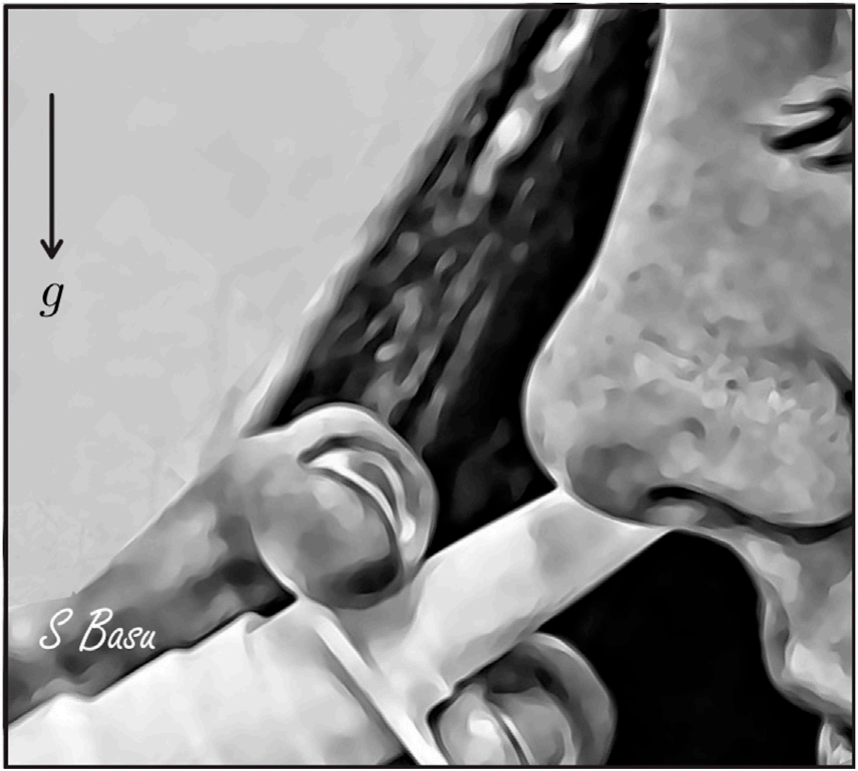
A demonstrative artistic rendering for the side view during “Improved Use” (IU) protocol, outlining how to ideally hold a spray bottle during intranasal administration with one’s head tilted slightly forward. *g* points to the direction of gravity. Rendering is courtesy of the corresponding author.

• *On inputs to targeted drug and device design–*With targeted delivery of pharmaceutical agents to the viral infection hot-spots in the posterior upper airway (e.g., at the nasopharynx) a clear challenge (Shah et al., 2014; Basu S. et al., 2020; Suman, 2021), the experimentally-validated findings from this study point to the droplet size range of ∼ 7–17 *μ*m as being the most effective at maximizing the sprayed and inhaled percentage deposition at the clinical upper airway target site for SARS-like infections. While it is *admittedly* challenging for today’s spray devices to consistently generate droplets/aerosols that small, the information from the current study can readily be used to inform the design of next-generation intranasal drug formulations, along with novel spray devices and atomizers. Such devices could be designed to maximize spray deposition within the ‘sweet spot’ described above. The iterative design of these devices is likely to involve engineering physical attributes of the spray device (for example, through adjusting nozzle sizes and pressure drops, adding baffles, and in general, by modifying the pump, actuator or formulation) to generate the required droplet sizes. Notably, the work described here not only provides a practical set of guidelines for device developers, but also provides an *in silico* platform for rapid iteration of device design (as the experimentally measured distribution of particle sizes generated by device prototypes can be rapidly evaluated for their deposition performance at the desired target site). In this context, the reader should also note that droplets and aerosols smaller than 10 *μ*m tend to often bypass the nose and deposit in the lungs. The process is dictated by the low inertia of the particulates which are consequently efficiently swept downstream into the lower airway by the inhaled airflow streamlines on which they are embedded. From a clinical translation perspective, this may warrant requests for safety studies from the regulatory bodies for toxicological assessment if significant lung deposition via the nose is demonstrated.
• *On inputs for effective spray usage strategies–*The significant 2 orders-of-magnitude improvement (see Figure 9) in nasopharyngeal delivery of intranasally sprayed drugs with the new IU protocol, over the typically recommended CU protocol, clearly warrants a revisit of the standard usage instructions for existing nasal spray products. While Section 2.3 lays out the criteria for *in silico* determination of the IU direction1; in ordinary language: the user can replicate the IU protocol by holding the spray nozzle as horizontally as possible at the nostril, with a slight tilt toward the cheeks. See Figure 10 for a sample pictorial demonstration. The results also hint at the utility potential of a device fitted with a bent nozzle.
• *On caveats regarding the tested droplet size range–*The tested droplet diameter range was 1–24 *μ*m. While extracting the droplet sizes that would correspond to at least 2% nasopharyngeal deposition from a cluster of 3,000 monodispersed droplets of each size, three of the eight test cases (i.e., the IU protocol data for left and right nostril administration in two subjects under two inhalation rates) led to 24 *μ*m as the maximum limit of such sizes; see Figures 6, 8. While that may justifiably raise the question on what happens if we consider droplets that are sized bigger than 24 *μ*m, the focus of this study has been to determine a common droplet size range that would be generically robust to inhaled airflow conditions and user subjectivities. Consequently, we did not track the bigger droplets which tend to deposit mostly anteriorly, owing to the inertia-dominated initial phase of their trajectories when injected out of the nozzle; see our earlier publication (Basu S. et al., 2020) for an extensive related discussion. Also, as a side-note to this, it is relevant to consider that administered droplets, under nebulized conditions in the same two test subjects and with comparable material density (1.3 g/mL), had resulted in an ideal size range of 2.5–19 *μ*m (i.e., comfortably smaller than 24 *μ*m) for at least 5% targeted nasopharyngeal deposition; see another of our earlier publications (Basu, 2021) for details.
• *On the limitations of respiratory flow modeling–*Realistic modeling of mucociliary transport along the morphologically complex airway cavity constitutes a significant open question in the domain of respiratory transport mechanics (Ford et al., 2021; Rajendran and Banerjee, 2021; Sekaran, 2021). In this study, we have implemented state-of-the-art algorithms to identify the droplet sizes that are efficient at *direct* nasopharyngeal delivery, under the impact of inhaled airflow when sprayed into the intranasal space. However, a substantial caveat lies in what happens to the larger droplets that happen to deposit along the anterior parts of the airway. Quantifying their mucus-driven downstream transport mechanics and correlating that with the therapeutic efficacy of the drug solutes when they reach the posterior clinical target sites poses a vital translational challenge, which needs to be addressed by the interdisciplinary scientific community in future.
• *On caveats related to the droplet transport modeling–*The Lagrangian particle transport scheme used to track the sprayed droplets is one-way momentum coupled with the continuous ambient airflow field. Additionally, the droplet tracking model ignores evaporation effects on the droplet constituents and any impact from the liquid wall films.
• First, in reality, momentum transfer from the nasal spray droplets to the surrounding fluid phase may indeed affect droplet motion and influence the nasal deposition patterns (Kolanjiyil et al., 2022). However, the one-way coupling approach for regional deposition prediction, apart from being computationally inexpensive, has been validated experimentally through multiple studies, both by us (Basu S. et al., 2020) and others (Inthavong et al., 2008; Feng et al., 2017; Zhao et al., 2021). Secondly, the droplet evaporation effects, while important for tracking the slow drug delivery process inside the lower airway along the branched bronchial pathways, could be considered negligible for drug delivery to sites in the upper respiratory tract, such as the nasopharynx. The time scale for sprayed droplet transport for direct nasopharyngeal deposition is merely on the order of $O\left(10^{-1}\right)$ s (Basu et al., 2018). With the scale at least 2 – 3 orders smaller than the evaporation time scale for a small droplet (Nguyen et al., 2012; Zang et al., 2019; Chatterjee et al., 2021), we argue that the inclusion of evaporative effects in the numerical scheme will have trivial impact on the direct deposition predictions at the nasopharynx. Finally, the non-consideration of the airway surface liquid film is a key limitation and a long-standing challenge, given the complex non-Newtonian rheology of the mucosal substrate (Lai et al., 2009). We will address this caveat in our future studies–especially to answer specific relevant questions, such as: (a) how long does a drug droplet stay at the target site before being swept downstream? (b) is the time scale from (a) sufficiently long for pharmaceutically effective tissue-level penetration of the drug solutes? and (c) what is the realistic nature of droplet dispersion and surface coverage over the liquid wall film?
• *On the constraints posed by the reconstructed in silico geometries–*The CT-based anatomically realistic reconstructions, while accurately replicating the topological convolutions implicit in a real tortuous respiratory cavity (Yuk et al., 2022; 2023), still come with the caveat of containing structurally rigid airway walls. However, though the rigidity of the walls (intended to mimic the internal tissue surfaces and cartilages) is somewhat unrealistic, the time-scale of inhaled transport is on the order of 10^−1^ s (Basu et al., 2018) and the idealization could be considered a mechanistically feasible assumption that is sufficient to extract the fundamental nuances underlying such physiologically complex transport processes.
• *On the usability of the findings despite the small test cohort–*The goal of our study was to design and test an improved protocol for administration of nasal sprays that is robust to person-to-person variation. Notably, within the geometries tested, the effect size observed by us (improved deposition efficiency) was two orders of magnitude. A key limitation of our findings is the restricted sample size of only two main test subjects (i.e., Subjects 1 and 2). However, the congruity in targeted delivery improvement (see Section 3.5; Figure 9) in a randomly-selected different subject (named as Subject 3) bodes well for the general extensibility of the essential findings to a wider cohort. The large observed effect size represents an encouraging preliminary finding, as we test our approach for generalizability and robustness to inter-individual variability
• *On toxicity evaluation–*Any new formulation or drug delivery device that might attempt to replicate the improved targeted deposition at intranasal sites, based on the current findings, will essentially constitute a surface contacting mechanism with limited duration contact. For determination of the usage safety levels, such a development will also require biocompatibility testing of the device, including a check of three basic biocompatibility end-points (*viz.* cytotoxicity, irritation (Basu et al., 2022b), sensitization) per the FDA guidance (ISO, 2009; FDA, 2022), by providing test data and/or relevant justification (e.g., history of clinical use for the same device).
• *On patient comfort and practicality of the IU protocol–*We have run a recent parallel study (Basu et al., 2022b) for assessing the human factors, e.g., the comfort levels, associated with spray placement protocols that are similar to the IU protocol proposed here and while using an open-angle swirling jet atomizer (GentleMist^®^; Dr. Ferrer Biopharma, Hallandale Beach, Florida). Evaluation feedback collected from a cohort of 13 healthy volunteers shows that the IU-like protocol offered a more gentle and soothing delivery experience, with less impact pressure. Also, 60% of participants reported that the CU technique caused painful irritation. In context to the practicality of the IU spray placement, the reader should additionally note that the physical experiments, results of which are outlined in Figure 9, were performed in a soft solid 3D-printed anatomic cast that replicated the pliability characteristics of real nasal tissues.

### 4.1 The main takeaways

Our conclusions can be viewed in the light of two different scenarios: (1) how can we achieve better target-site coverage with existing sprays, and (2) what are the insights here for the design of improved spray devices in the future? Intranasal sprays could represent a useful administration strategy for nasal hygiene products, antiviral agents, and even vaccines (Afkhami et al., 2022; Axe, 2022; Mao et al., 2022)–for respiratory pathogens that would first trigger an upper airway infection, such as SARS-CoV-2. In this study, we have implemented experimentally verified computational simulations of respiratory transport and drug delivery to illustrate that simple tweaks to the nasal spray direction can result in vastly improved drug deposition at the critical viral infection sites inside the nose. More specifically, even with prevalent realistic droplet size distributions as found in administered shots of over-the-counter spray products–the delivered dose at the target infective site (i.e., the nasopharynx) registers an improvement of approximately 2 orders-of-magnitude with the new IU protocol.

The proposed IU nasal spray protocol (see Figure 2 in the introduction, along with Figure 10 here; Figures 3, 4 in Section 2) is easy-to-replicate and has been verified to be robust to small perturbations that may stem from user subjectivities. Additionally, we found the droplet size range of ∼ 7–17 *μ*m to be most efficient at facilitating direct delivery of intranasally sprayed particulates at the nasopharynx, which is the dominant infection trigger zone for respiratory viruses. The findings hold the potential to help develop increasingly effective intranasal pharmaceutic formulations, along with refined designs for nasal drug delivery devices and atomizers.

## Data Availability

The original contributions presented in the study are included in the article/supplementary materials and further inquiries can be directed to the corresponding author.
